# Study on the Prevalence of Severe Anemia among Non-Pregnant Women of Reproductive Age in Rural China: A Large Population-Based Cross-Sectional Study

**DOI:** 10.3390/nu9121298

**Published:** 2017-11-28

**Authors:** Qiuyue Ma, Shikun Zhang, Jue Liu, Qiaomei Wang, Haiping Shen, Yiping Zhang, Min Liu

**Affiliations:** 1 Department of Epidemiology and Biostatistics, School of Public Health, Peking University, 100191 Beijing, China; mqyue10@163.com (Q.M.); liujue7@163.com (J.L.); 2 Department of Maternal and Child Health, National Health and Family Planning Commission of the PRC, 100190 Beijing, China; shikun_zhang1121@163.com (S.Z.); qiaomei_w@163.com (Q.W.); shenhaiping68@126.com (H.S.); yiping791129@126.com (Y.Z.)

**Keywords:** severe anemia, prevalence, non-pregnant women, China, rural areas

## Abstract

Globally, severe anemia impacts millions of non-pregnant women. However, studies on the prevalence of severe anemia through large epidemiologic surveys among non-pregnant women have been scarce in China. In this study, we aimed to study the prevalence of severe anemia and its determinants among non-pregnant women living in rural areas of China. Data were gathered for 712,101 non-pregnant women aged between 21 and 49 years who attended the 2012 National Free Preconception Health Examination Project. Severe anemia in non-pregnant women was defined as a hemoglobin (Hb) concentration lower than 80 g/L. Associated factors were analyzed using univariate and multivariate logistic regression methods. Out of the 712,101 non-pregnant women living in the rural areas of China, 1728 suffered from severe anemia, with a prevalence of 0.24% (95% confidence interval (CI): 0.23–0.25%). Results from the multivariable logistic regression showed that elderly (adjusted odds ratio (aOR) = 3.08), living in the northwest region (aOR = 2.88), having a history of anemia (aOR = 5.76), with heavy menstrual blood loss (aOR = 1.84), and with a history of using an intra-uterine device (aOR = 1.47) etc., were independent determinants for women with severe anemia in rural China. The prevalence of severe anemia among Chinese non-pregnant women living in the rural areas was lower than the reported global prevalence. Prevention and intervention programs for severe anemia are required among non-pregnant women of reproductive age in the rural areas of China.

## 1. Introduction

Anemia is of public health concern that affects countries with low, middle, or high income [1]. It is especially prevalent in women of reproductive age [2]. Anemia is defined as a low blood hemoglobin (Hb) concentration. The causes of anemia include iron and other micronutrient deficiencies, acute and chronic infections, inherited or acquired disorders, etc. [1]. Patients with anemia present similar clinical symptoms such as fatigue, breathlessness, dizziness, and headache [3]. Anemia also increases the susceptibility to different kinds of infections and impairs the work capacity [4]. Severity of symptom caused by anemia is paralleled with the severity of anemia [5]. Severe anemia may predispose to infection and heart failure [6], while severe anemia during pregnancy may significantly contribute to both maternal mortality and morbidity [7]. Severe maternal anemia carries a significant risk of hemorrhage and infection in mothers [8], reduces the resistance to blood loss causing maternal death [2], and place women at higher risk of death during delivery and the period following childbirth [9]. Maternal anemia may also increase the risk of adverse pregnancy outcomes, such as preterm birth, low birth weight, small size for gestational age infants, perinatal death, and anemia in infancy [10,11,12,13,14].

Most of the previous studies were focused on the prevalence and determinants of anemia among pregnant women [3,6,12], but few examined severe anemia prevalence in non-pregnant women. The World Health Organization (WHO) reported that 58% of the pregnant women with anemia were also anemic before being pregnant [2]. Anemia appeared as an important public health problem throughout the world, particularly in developing countries [15,16]. Globally, the overall prevalence of severe anemia among non-pregnant women of reproductive age was 1.1%, affecting 19 million non-pregnant women [13]. Prevention of anemia in non-pregnant women could improve the health status of the pregnant women, eventually contributing to the reduction of both maternal and perinatal mortality.

As a developing country, China should pay greater attention to severe anemia in Chinese women of reproductive age. According to a WHO report in 2011, the prevalence of severe anemia among Chinese non-pregnant women was 0.3%, lower than both the global prevalence (1.1%) and the prevalence in Asia (1.3%) [1]. However, there were few studies assessing the prevalence of severe anemia among non-pregnant women in the country, and most of the criteria they used were different from the ones recommended by WHO (Hb concentration lower than 80 g/L [17]). Only one study followed the WHO criteria, which was conducted among non-pregnant and not breastfeeding women in 1998, covering 11 provinces of China, showing the prevalence of severe anemia as 2.1% [18]. No large population-based study focusing on the prevalence of severe anemia among Chinese reproductive age women have been carried out in recent years.

In this study, the original data we used was from the 2012 National Free Preconception Health Examination Project (NFPHEP). The criteria we used to estimate the prevalence of severe anemia in Chinese rural non-pregnant women was following WHO recommendation. In this paper, the estimated prevalence on severe anemia prevalence among non-pregnant women of reproductive age in rural China had been updated.

## 2. Materials and Methods

### 2.1. Study Design

This was a population-based cross-sectional study in which involved 712,101 non-pregnant women aged 21–49 years who attended the NFPHEP between 1 January 2012 and 31 December 2012. Participants were from 193 counties in 30 provinces; from all over the country. All the participants were given written informed consent forms before enrollment. Data regarding the Hb concentration was obtained from the laboratory tests. Hb concentration was measured using the cyanomethemoglobin method by trained medical staff. A standardized questionnaire was used by local health workers to collect data on demographic characteristics (including age; education levels; occupation; ethnic origins and address of residence). Histories on using contraceptive methods and reproduction (including histories on using intra-uterine devices (IUD) pregnancy; miscarriages and the number of pregnancies); health status (including history of anemia; menstrual blood loss; body mass index (BMI)); and meat and egg eating habits of the participants were also collected. Both detailed design and quality assessment on NFPHEP were stated in other articles elsewhere [19,20].

In the questionnaire, we asked the participants about their nutritional behavior, whether they ate meat and eggs in their regular lives. If the answer was “yes”, we then considered they were meat and egg eaters. During the physical examinations, measurements of height and weight of participants were performed by qualified and trained physicians. BMI was calculated as weight in kilograms divided by height in meters squared (kg/m^2^).

According to the administrative divisions, the 30 provinces were divided into six regions, as follows: north region (including Beijing, Tianjin, Hebei, Shanxi, Inner Mongolia), northeast region (including Liaoning, Jilin, Heilongjiang), east region (including Shanghai, Jiangsu, Zhejiang, Anhui, Fujian, Jiangxi, Shandong), central south region (including Henan, Hubei, Hunan, Guangdong, Guangxi, Hainan), southwest region (including Chongqing, Sichuan, Guizhou, Yunnan), and northwest region (including Shaanxi, Gansu, Qinghai, Ningxia, Xinjiang). According to the age distributions, the participants were divided into six groups as follows: 21–24 years, 25–29 years, 30–34 years, 35–39 years, 40–44 years, and 45–49 years old. Again, according to the education levels, the participants were divided into four groups as follows: primary school or below, junior high school, senior high school, and college or higher. Other groupings would include: occupation (farmers, workers, and others), ethnic origin (Han and others), self-reported menstrual blood loss (minor, medium, or heavy), BMI (lower than 18.5, 18.5–24, higher than 24), and number of pregnancies (0, 1, ≥2).

### 2.2. Case Definition of Severe Anemia

We followed the definition of severe anemia as the thresholds for Hb concentration of non-pregnant women as lower than 80 g/L [17], recommended by the WHO. We also adjusted the thresholds for altitude since it was related to Hb concentration. Adjustments of severe anemia thresholds for people living at altitudes higher than 1000 m were recommended by the WHO: thresholds plus 2 g/L for people living at altitudes between 1000 m and 1500 m; plus 5 g/L for those living at altitudes between 1500 m and 2000 m; plus 8 g/L at 2000–2500 m; plus 13 g/L at 2500–3000 m; plus 19 g/L at 3000–3500 m; plus 27 g/L at 3500–4000 m; 35 g/L at 4000–4500 m; and plus 45 g/L at altitudes higher than 4500 m [17].

### 2.3. Methods Used for Statistical Analysis

Proportions were used to describe data related to demographic characteristics, contraceptive method, reproduction, and the health status of the participants. The prevalence of severe anemia was estimated using the percentage and 95% confidence interval (CI). Univariate and multivariate logistic regression methods were used to analyze determinants related to severe anemia. Data was analyzed under the stratification of the histories of using IUD and pregnancy. Analysis by the number of pregnancies was also performed. We considered two-tailed *p* values of lower than 0.05 to be statistically significant. All methods used for analyses were performed with SPSS Statistics for Windows, Version 19 (IBM Corp, Armonk, NY, USA).

### 2.4. Ethical Approval

All subjects gave their informed consent for inclusion before they participated in the study. The study was conducted in accordance with the Declaration of Helsinki, and the study was approved by Institutional Review Board of Chinese Association of Maternal and Child Health Studies (Project identification code AMCHS-2014-6).

## 3. Results

The mean age of all women who participated in this study was 27.37 ± 4.66 years. Demographic characteristics of the participants are as follows: 532,246 (74.74%) were between 21 and 29 years; 475,660 (66.79%) had an education level of junior high school or below; 518,803 (72.86%) were farmers; 640,820 (89.99%) were of Han ethnicity; and 246,735 (34.65%) were from the central south region (Table 1).

Of the 712,101 non-pregnant women, 1728 suffered severe anemia, with a prevalence of 0.24% (95% CI: 0.23–0.25%). The prevalence was significantly higher in those 45–49 years old (0.92%) than in other age groups (0.20–0.63%). The prevalence of severe anemia was significantly lower in women with education levels of junior high school (0.29%), senior high school (0.15%), college or higher (0.10%), but appeared the highest in women who only received primary or even less education (0.45%). The prevalence seen in farmers (0.28%) was significantly higher than in workers (0.14%) or in other occupations (0.13%). The prevalence of women with other ethnic origins (0.47%) was significantly higher than those in Han Chinese (0.22%). Women who lived in the northwest region had the highest prevalence (0.51%) than those living in other regions (0.15–0.32%, Table 1).

The prevalence rates of women appeared higher among those with history of using IUD (0.49%) and pregnancy (0.34%) than those without (0.20%, 0.16%, respectively). The prevalence of women with the history of miscarriage (0.27%) was higher than those without (0.24%), but with no significant difference. Women having had a history of anemia showed significantly higher prevalence than those without (1.09%, 0.23%, respectively). Compared with groups under minor or medium menstrual blood loss, the prevalence of severe anemia in women with heavy menstrual blood loss appeared significantly higher. Women who had a BMI between 18.5 and 24, or enjoyed eating meat and eggs, had higher prevalence than those in other opposite groups, but with no significant differences (Table 2).

Through multivariate logistic regression analysis, factors as: being elderly (aOR = 3.08), being other ethnicities (aOR = 1.28), living in the northwest regions (aOR = 2.88), histories of using IUD (aOR = 1.47), pregnancy (aOR = 1.38), anemia (aOR = 5.76), and heavy menstrual blood loss (aOR = 1.84), etc., were all associated with the prevalence of severe anemia. Women aged 45–49 years were more likely to have severe anemia than women aged 21–24 years (aOR = 3.08, 95% CI: 2.06–4.60). Women with education levels of primary school or lower and farmers had higher risks of having severe anemia. Women of other ethnicities were 1.28 times more likely of having severe anemia than those of Han ethnicity. Compared to the southwest region, women who lived in the northwest region were more likely to be severely anemic (aOR = 2.88, 95% CI: 2.37–3.51). A history of using IUD or being pregnant increased the risk of suffering severe anemia by 1.47 and 1.38 times, respectively. A history of miscarriage showed an inverse relationship with severe anemia (aOR = 0.85, 95% CI: 0.74–0.98). A history of ever being anemic increased the risk of severe anemia by 5.76 times. Women with heavy menstrual blood loss were 1.84 times more likely to be severely anemic to women with only minor blood loss. The risk of severe anemia in women who had a BMI ≥ 24 was 24% lower, compared to women who had a BMI below 18.5. In our study, meat and egg eating habits did not seem to be linked to the increased risk of severe anemia (Table 1 and Table 2).

**Figure 1:**
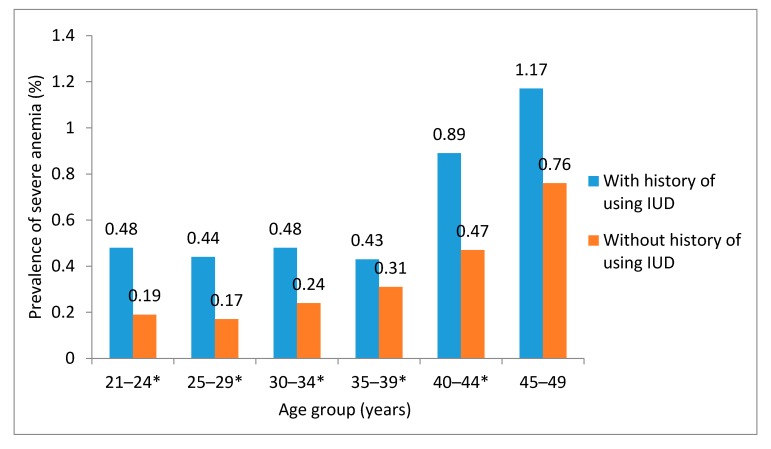
Age-specific prevalence rates of severe anemia in different histories of using intra-uterine device (IUD) in 2012, rural China. * The age group with significant difference in severe anemia prevalence between women with a history of using IUD and those without.

We analyzed the prevalence of severe anemia stratified by IUD use. Data showed that the distributions of prevalence rates in different age, education level, region of residency, and history of anemia, were similar to the distributions in all women, regardless of having the history of using the IUD. Women with a history of using IUD showed higher prevalence of severe anemia than those without, and the differences were significant in 21–24, 25–29, 30–34, 35–39, and 40–44 year old age groups (Figure 1). Having a history of pregnancy appeared as a determinant for severe anemia in women without the history of using IUD (aOR = 1.39, 95% CI: 1.19–1.61), but not otherwise. Having a history of miscarriage was not associated with severe anemia in women with a history of using IUD. However, it was a protective factor for women without the history of using IUD (aOR = 0.77, 95% CI: 0.64–0.93). Women with heavy menstrual blood loss and without the history of using IUD had an increased risk of developing severe anemia (aOR = 1.80, 95% CI: 1.14–2.83, Table 3).

**Figure 2:**
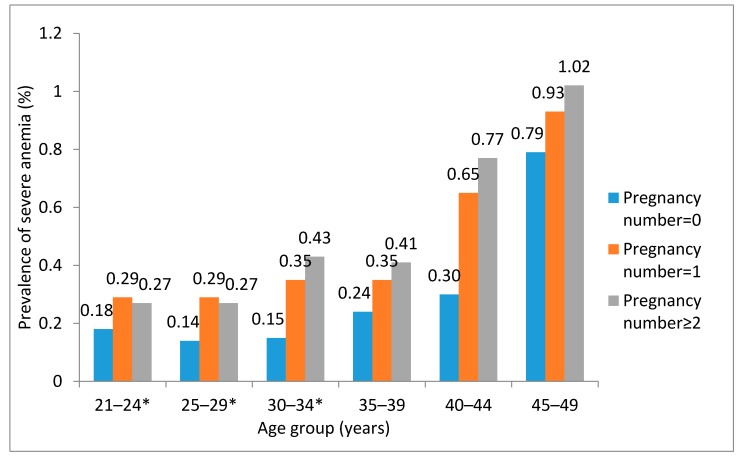
Age-specific prevalence rates of severe anemia in women with different pregnancy numbers in 2012, rural China. * The age group with significant difference in severe anemia prevalence in different pregnancy numbers.

Analysis stratified by history of pregnancy was also performed. We found that women with a history of pregnancy had higher prevalence of severe anemia than women who were without such a history. Women aged 45–49 years had a higher risk of suffering severe anemia than other age groups, regardless of the history of pregnancy (Table 4). Furthermore, we conducted an analysis stratified by the number of pregnancies. The result showed that women with different pregnancy numbers had different prevalences of severe anemia. The prevalence was significantly different in 21–24, 25–29, and 30–34 year old age groups (Figure 2).

## 4. Discussion

Anemia in non-pregnant women is a global public health concern. In 2011, WHO reported that the global overall prevalence of severe anemia among non-pregnant women was 1.1%, with the highest seen in Africa (1.6%) and the lowest in Northern America (0.2%) [1]. The prevalence of severe anemia among non-pregnant women in Asia was 1.3% [1]. In our study, the prevalence of severe anemia prevalence appeared as 0.24% in Chinese non-pregnant women living in rural areas. This overall prevalence was lower than that reported in Asia and seemed consistent in China (0.3%), as estimated by the WHO [1].

Previous studies noticed that demographic characteristics, such as age and levels of education, were associated with the prevalence of severe anemia [21,22]. An American study discovered that the rates on both anemia and moderate-severe anemia increased bimodally, with peaks seen in the 40–49 and 80–85 age groups [21]. Data from a study on anemia prevalence among Chinese non-pregnant women conducted in 11 provinces in 1998 discovered that the highest prevalence was seen among rural women aged 40–44 years (2.0%) than from the other age groups (0.3–1.4%) [18]. In our study, the result was similar with the above-mentioned study. We also noticed that the prevalence was higher in 40–49 year olds (0.63–0.92%) than in other age groups (0.20–0.35%). Our results indicated that older women of reproductive age had a higher risk of developing severe anemia.

Previous study also showed that the levels of education were related to healthcare seeking behavior [23], as well as the knowledge of nutrition of the women. Women are often responsible for producing and preparing food for the household, so their knowledge—or lack thereof—about nutrition can affect the health and nutritional status of the entire family [9]. Our study showed that the prevalence of severe anemia was seen as the highest among women with an education level of primary school or below, and appeared the lowest among women with an education level of college or higher. This result was consistent with previous studies [16,22,23]. Bentley et al. [16] reported a decreased risk of severe anemia among ever-married women with high school or higher education level in India. A study in Bangladesh reported that a lack of education was an important factor related to the prevalence of anemia among Bangladeshi women, and the authors believed that the increase in the education level would likely bring about a decline in the prevalence of anemia [22]. Wilunda et al. [23] found that primary school education reduced the risk of severe anemia by about 20%, compared with those having no education at all.

In our study, the prevalence of severe anemia in farmers was significantly higher than in workers and other occupations, which might be related to the low socioeconomic status of farmers, and which represented insufficient diet, limited education, inadequate access to health services, and delayed diagnosis and treatment [16,24] in this population. Meanwhile, farmers were exposed to occupational hazards, including extreme weather conditions, exposure to dangerous chemicals, schistosomiasis, and hookworm infections [5,24], which may lead them to suffer from severe anemia. The reduced work capacity caused by anemia was a burden on the rural Chinese women who were responsible for both domestic and field labor work. Low work capacity due to severe anemia often negatively affected household maintenance and income [5,25]. In different regions, the prevalence of severe anemia among women living in the northwest was the highest (0.51%), while it was lowest among women in the central south (0.15%). The geographic differences in severe anemia prevalence rates might correspond to the inequality of income and food availability in rural areas.

Histories on the use of contraceptive methods, reproduction, and health status were also associated with the prevalence of severe anemia. Approximately 50% of the anemic cases were considered to be related to iron deficiency [1]. Again, IUD use, menstrual blood loss, and pregnancy can cause extra iron loss in women. Iron deficiency anemia (IDA) arises when the body’s loss of iron is insufficient to fully support red cell production [26]. We found that the prevalence of severe anemia in women who self-reported having a history of anemia was significantly higher than women without having the history on anemia (OR = 5.76). This result indicated that anemic women who had not received effective prevention or treatment after the diagnosis was made usually ended up with a worsening status of anemia.

IUD are recognized as a modern effective contraceptive method, with less than 1% failure [27]. In China, a report in 2007 stated that there were 114 million women who use IUD, accounting for 49.79% of all the contraceptive methods being used [28]. The main side effect of IUD use was noted that it could increase the menstrual blood loss and IDA [29]. In our study, the risk of severe anemia among women with history of IUD use was 1.47 times higher than those without, and this trend was found similar in different age groups. Meanwhile, women aged 45–49 years had a 2.90 times higher risk for severe anemia than those aged 21–24 years in the IUD-using group. Our findings indicated that women should take care of their own health status, especially for elder women, and choosing the IUD with caution.

Menstruation was an important factor which contributed to a negative iron balance in women [30]. We found that heavy menstrual blood loss was a determinant related to severe anemia in women at all ages. Stratified analysis on IUD use revealed that in women without the history of IUD use, heavy blood loss appeared as a determinant of severe anemia (OR = 1.80). This finding indicated that blood loss should be highly concerned among women without the history of IUD use. In fact, every non-pregnant woman would lose 25–30 mL extra blood each month [2], which should prompt all women of reproductive age to monitor their iron status regularly.

Parity was found to be negatively associated with Hb concentration in previous study [31]. Hb concentration was noted to be lower in women with higher parity, which was a determinant for IDA. In our study, after adjusting the history of pregnancy to replace parity, data showed that the result was similar. The risk of severe anemia in women who had a history of pregnancy was 1.38 times to those who did not, and this trend was similar in different age groups. However, history of miscarriage did not seem to be a risk factor for severe anemia in women with a history of using an IUD, but appeared as a protective factor for women without such a history. This result suggested that the association between miscarriage and severe anemia requires further study.

Compared with non-heme iron, absorption of heme iron does not require binding proteins [32], so its uptake is easy for human beings. For non-vegetarians, heme iron, especially those from red meat, such as beef and lamb, contribute at least 40% of the total iron absorbed [33]. Therefore, women living in the rural areas of China who consume mostly grains and vegetables would absorb less iron from their diet. However, we did not find that the habit of eating meat and eggs was a factor associated with severe anemia, with the postulation that severe anemia was mostly related to the decrease of red cell production [5]. Less iron intake from the diet might lead to mild, rather than severe, anemia. Additionally, there were few women who were not meat and egg eaters (1.04%) in our study, which might interfere with the results.

To our knowledge, this study was the largest population-based one on severe anemia that focused on non-pregnant women in rural China. Prior to our study, there were only two surveys regarding national nutrition status, conducted in 2002 and 2010 [34,35] that mentioned the overall prevalence on anemia rather than on severe anemia, among women of reproductive age. A small sample-sized study is difficult to assess the prevalence of severe anemia, which is a rare disease in nature. The sample size of our study was large enough to have covered 30 provinces, 193 counties of China, with a total population size as large as 712,101. The definition of severe anemia was under the criteria set by WHO, enabling our result to compare with other study of the same kind.

Limitations in our study include the following: the inclusion of our sample only involved women who were not pregnant, but planned to get pregnant in six months, from rural China, leaving the prevalence not representative for all women of reproductive age in the country. There were few severe anemia cases in some subgroups, which was a limitation for the results we reported. Data regarding time was not mentioned on IUD use and histories of pregnancy or miscarriage, which limited the interpretation of the association between severe anemia and these factors. There was potential bias due to the inclusion of anemia caused by specific etiology, such as genetic disorders.

## 5. Conclusions

In conclusion, the prevalence of severe anemia among non-pregnant women living in rural areas in China was lower than the global prevalence, despite the fact that it was seen as higher in some particular groups. Women who were elderly, living in the northwest region, with low education levels, being farmers, having had histories of anemia or ever using IUD, and with heavy menstrual blood loss, etc., were under higher risk for severe anemia. With the limited medical resources in rural areas, women with higher risk of severe anemia should have priority to be paid close attention. Effective strategies on prevention and management of anemia in non-pregnant women can improve the health status of these women prior to, or during, pregnancy, eventually contributing to the reduction on adverse pregnancy outcomes, and maternal and perinatal mortality.

## Figures and Tables

**Table 1:** Demographic characteristics and severe anemia prevalence among non-pregnant women of reproductive age in 2012, rural China.

	Number	%	Severe Anemia (*n*)	Prevalence of Severe Anemia (% (95% CI))	cOR (95% CI)	aOR ^a^ (95% CI)
**All participants**	712,101	100.00	1728	0.24 (0.23–0.25)	_	_
**Age group, years**						
21–24	216,573	30.41	448	0.21 (0.19–0.23)	1.00	1.00
25–29	315,673	44.33	636	0.20 (0.19–0.22)	0.97 (0.86–1.10)	0.94 (0.82–1.07)
30–34	120,336	16.90	372	0.31 (0.28–0.34)	1.50 (1.30–1.72)	1.19 (1.02–1.38)
35–39	40,199	5.65	141	0.35 (0.29–0.41)	1.70 (1.41–2.05)	1.10 (0.89–1.36)
40–44	16,155	2.27	102	0.63 (0.51–0.75)	3.07 (2.47–3.80)	2.16 (1.71–2.73)
45–49	3165	0.44	29	0.92 (0.58–1.25)	4.46 (3.06–6.51)	3.08 (2.07–4.60)
**Education**						
Primary school or below	31,150	4.37	140	0.45 (0.38–0.52)	1.00	1.00
Junior high school	444,510	62.42	1287	0.29 (0.27–0.31)	0.64 (0.54–0.77)	0.78 (0.65–0.94)
Senior high school	131,986	18.53	192	0.15 (0.12–0.17)	0.32 (0.26–0.40)	0.54 (0.43–0.69)
College or higher	92,826	13.04	94	0.10 (0.08–0.12)	0.23 (0.17–0.29)	0.43 (0.32–0.58)
Not available	11,629	1.63	_	_	_	_
**Occupation**						
Farmers	518,803	72.86	1473	0.28 (0.27–0.30)	1.00	1.00
Workers	64,527	9.06	88	0.14 (0.11–0.16)	0.48 (0.39–0.60)	0.65 (0.52–0.82)
Others	113,606	15.95	149	0.13 (0.11–0.15)	0.46 (0.39–0.55)	0.76 (0.62–0.93)
Not available	15,165	2.13	_	_	_	_
**Ethnic origin**						
Han	640,820	89.99	1425	0.22 (0.21–0.23)	1.00	1.00
Others	62,262	8.74	293	0.47 (0.42–0.52)	2.12 (1.87–2.41)	1.28 (1.11–1.49)
Not available	9019	1.27	_	_	_	_
**Region**						
Southwest	92,598	13.00	157	0.17 (0.14–0.20)	1.00	1.00
Central south	246,735	34.65	382	0.15 (0.14–0.17)	0.91 (0.76–1.10)	1.16 (0.95–1.42)
North	87,890	12.34	220	0.25 (0.22–0.28)	1.48 (1.20–1.81)	1.65 (1.32–2.06)
Northeast	47,767	6.71	153	0.32 (0.27–0.37)	1.89 (1.51–2.36)	1.81 (1.42–2.30)
East	151,866	21.33	381	0.25 (0.23–0.28)	1.48 (1.23–1.78)	1.84 (1.51–2.26)
Northwest	85,245	11.97	435	0.51 (0.46–0.56)	3.02 (2.52–3.63)	2.88 (2.37–3.51)

^a^ Adjusted for age, education, occupation, ethnic origin, region, history of using intra-uterine device (IUD), histories on pregnancy or miscarriage, history on anemia, menstrual blood loss, body mass index (BMI), and meat and egg eating habits. CI: confidence interval; cOR: crude odds ratio; aOR: adjusted odds ratio.

**Table 2:** Histories on using contraceptive methods, reproduction, health status, and severe anemia prevalence among non-pregnant women of reproductive age in 2012, rural China.

	Number	%	Severe Anemia (*n*)	Prevalence of Severe Anemia (% (95% CI))	cOR (95% CI)	aOR ^a^ (95% CI)
**All participants**	712,101	100.00	1728	0.24 (0.23–0.25)	_	_
**History of using IUD**						
No	601,121	84.42	1186	0.20 (0.19–0.21)	1.00	1.00
Yes	110,980	15.58	542	0.49 (0.45–0.53)	2.48 (2.24–2.75)	1.47 (1.30–1.67)
**History on pregnancy**						
No	388,597	54.57	628	0.16 (0.15–0.17)	1.00	1.00
Yes	319,189	44.82	1096	0.34 (0.32–0.36)	2.13 (1.93–2.35)	1.38 (1.21–1.59)
Not available	4315	0.61	_	_	_	_
**History on miscarriage**						
No	598,045	83.98	1424	0.24 (0.23–0.25)	1.00	1.00
Yes	114,056	16.02	304	0.27 (0.24–0.30)	1.12 (0.99–1.27)	0.85 (0.74–0.98)
**History on anemia**						
No	703,399	98.78	1633	0.23 (0.22–0.24)	1.00	1.00
Yes	8702	1.22	95	1.09 (0.87–1.31)	4.74 (3.85–5.84)	5.76 (4.63–7.18)
**Menstrual blood loss**						
Minor	20,033	2.81	36	0.18 (0.12–0.24)	1.00	1.00
Medium	661,682	92.92	1589	0.24 (0.23–0.25)	1.34 (0.96–1.86)	1.31 (0.94–1.83)
Heavy	24,023	3.37	95	0.40 (0.32–0.47)	2.21 (1.50–3.24)	1.84 (1.25–2.71)
Not available	6363	0.89	_	_	_	_
**BMI**						
<18.5	77,389	10.87	170	0.22 (0.19–0.25)	1.00	1.00
18.5–24	514,707	72.28	1274	0.25 (0.23–0.26)	1.13 (0.96–1.32)	0.93 (0.79–1.09)
≥24	77,308	10.86	186	0.24 (0.21–0.28)	1.10 (0.89–1.35)	0.76 (0.61–0.94)
Not available	42,697	6.00	_	_	_	_
**Meat and egg eaters**						
Yes	699,519	98.23	1700	0.24 (0.23–0.25)	1.00	1.00
No	7379	1.04	17	0.23 (0.12–0.34)	0.95 (0.59–1.53)	0.82 (0.47–1.41)
Not available	5203	0.73	_	_	_	_

^a^ Adjusted for age, education, occupation, ethnic origin, region, histories of using IUD, pregnancy, miscarriage, anemia, menstrual blood loss, BMI, meat and egg eating habit.

**Table 3:** Prevalence of severe anemia among non-pregnant women of reproductive age, stratified by history of using IUD in 2012, rural China.

	With History of Using IUD				Without History of Using IUD			
	Number	Severe Anemia, *n* (%)	cOR (95% CI)	aOR ^a^ (95% CI)	Number	Severe Anemia, *n* (%)	cOR (95% CI)	aOR ^a^ (95% CI)
**Age group, years**								
21–24	11,618	56 (0.48)	1.00	1.00	204,955	392 (0.19)	1.00	1.00
25–29	41,879	184 (0.44)	0.91 (0.68–1.23)	1.03 (0.75–1.41)	273,794	452 (0.17)	0.86 (0.75–0.99)	0.90 (0.78–1.04)
30–34	35,262	169 (0.48)	0.99 (0.73–1.35)	1.20 (0.87–1.65)	85,074	203 (0.24)	1.25 (1.05–1.48)	1.19 (0.99–1.43)
35–39	14,733	63 (0.43)	0.89 (0.62–1.27)	0.95 (0.64–1.40)	25,466	78 (0.31)	1.60 (1.26–2.05)	1.26 (0.96–1.65)
40–44	6293	56 (0.89)	1.85 (1.28–2.69)	2.41 (1.63–3.57)	9862	46 (0.47)	2.45 (1.80–3.32)	1.90 (1.36–2.65)
45–49	1195	14 (1.17)	2.45(1.36–4.41)	2.90 (1.55–5.41)	1970	15 (0.76)	4.00 (2.39–6.72)	3.23 (1.87–5.56)
**Education**								
Primary school or below	9079	66 (0.73)	1.00	1.00	22,071	74 (0.34)	1.00	1.00
Junior high school	88,897	442 (0.50)	0.68 (0.53–0.89)	0.72 (0.55–0.96)	355,613	845 (0.24)	0.71 (0.56–0.90)	0.82 (0.63–1.05)
Senior high school	9719	30 (0.31)	0.42 (0.27–0.65)	0.53 (0.34–0.84)	122,267	162 (0.13)	0.39 (0.30–0.52)	0.56 (0.42–0.75)
College or higher	2233	4 (0.18)	0.25(0.09–0.67)	0.32 (0.11–0.90)	90,593	90 (0.10)	0.30 (0.22–0.40)	0.46 (0.32–0.65)
**Occupation**								
Farmers	99,730	508 (0.51)	1.00	1.00	419,073	965 (0.23)	1.00	1.00
Workers	4799	15 (0.31)	0.61 (0.37,1.02)	0.71 (0.42–1.21)	59,728	73 (0.12)	0.53 (0.42–0.67)	0.63 (0.49–0.82)
Others	5042	17 (0.34)	0.66 (0.41,1.07)	0.89 (0.54–1.48)	108,564	132 (0.12)	0.53 (0.44–0.63)	0.73 (0.59–0.91)
**Ethnic origin**								
Han	90,262	408 (0.45)	1.00	1.00	550,558	1017 (0.18)	1.00	1.00
Others	20,359	134 (0.66)	1.46 (1.20–1.78)	1.21 (0.96–1.54)	41,903	159 (0.38)	2.06 (1.74–2.43)	1.34 (1.10–1.62)
**Region**								
Southwest	16,655	47 (0.28)	1.00	1.00	75,943	110 (0.14)	1.00	1.00
Central south	21,128	63 (0.30)	1.06 (0.72–1.54)	1.31 (0.87–1.97)	225,607	319 (0.14)	0.98 (0.79–1.21)	1.09 (0.86–1.38)
North	14,832	75 (0.51)	1.80 (1.25–2.59)	2.34 (1.57–3.49)	73,058	145 (0.20)	1.37 (1.07–1.76)	1.40 (1.07–1.83)
Northeast	11,141	69 (0.62)	2.20 (1.52–3.19)	2.62 (1.74–3.94)	36,626	84 (0.23)	1.59 (1.19–2.11)	1.44 (1.06–1.96)
East	28,914	127 (0.44)	1.56 (1.12–2.18)	2.11 (1.45–3.06)	122,952	254 (0.21)	1.43 (1.14–1.79)	1.74 (1.37–2.22)
Northwest	18,310	161 (0.88)	3.14 (2.26–4.34)	3.41 (2.40–4.85)	66,935	274 (0.41)	2.83 (2.27–3.54)	2.66 (2.10–3.38)
**History on pregnancy**								
No	2684	7 (0.26)	1.00	1.00	385,913	621 (0.16)	1.00	1.00
Yes	108,223	535 (0.49)	1.90(0.90–4.01)	1.55(0.73–3.28)	210,966	561 (0.27)	1.65 (1.48–1.86)	1.39 (1.19–1.61)
**History on miscarriage**								
No	84,429	416 (0.49)	1.00	1.00	513,616	1008 (0.20)	1.00	1.00
Yes	26,551	126 (0.47)	0.96 (0.79–1.18)	0.98(0.79–1.21)	87,505	178 (0.20)	1.04 (0.88–1.22)	0.77 (0.64–0.93)
**History on anemia**								
No	109,641	516 (0.47)	1.00	1.00	593,758	1117 (0.19)	1.00	1.00
Yes	1339	26 (1.94)	4.19(2.81–6.23)	4.74(3.13–7.17)	7363	69 (0.94)	5.02 (3.93–6.41)	6.33 (4.89–8.20)
**Menstrual blood loss**								
Minor	2497	7 (0.28)	1.00	1.00	17,536	29 (0.17)	1.00	1.00
Medium	102,324	497 (0.49)	1.74 (0.82–3.67)	1.81 (0.85–3.83)	559,358	1092 (0.20)	1.18 (0.82–1.71)	1.20 (0.83–1.74)
Heavy	5690	37 (0.65)	2.33 (1.04–5.23)	2.22 (0.98–5.02)	18,333	58 (0.32)	1.92 (1.23–2.99)	1.80 (1.14–2.83)

^a^ Adjusted for age, education, occupation, ethnic origin, residence of region, histories on pregnancy/miscarriage/anemia, menstrual blood loss/BMI/habit on eating meat and eggs.

**Table 4:** Prevalence of severe anemia in different age groups among non-pregnant women of reproductive age, stratified by the history of pregnancy in 2012, rural China.

Age Group, Years	With Pregnancy History				Without Pregnancy History			
	Number	Severe Anemia, *n* (%)	cOR (95% CI)	aOR ^a^ (95% CI)	Number	Severe Anemia, *n* (%)	cOR (95% CI)	aOR ^a^ (95% CI)
21–24	54,794	159 (0.29)	1.00	1.00	160,081	288 (0.18)	1.00	1.00
25–29	131,040	377 (0.29)	0.99 (0.82–1.19)	1.00 (0.82–1.22)	182,968	258 (0.14)	0.78 (0.66–0.93)	0.89 (0.74–1.06)
30–34	84,108	317 (0.38)	1.30 (1.07–1.57)	1.29 (1.05–1.57)	35,558	54 (0.15)	0.84 (0.63–1.13)	1.01 (0.74–1.36)
35–39	32,791	123 (0.38)	1.29 (1.02–1.64)	1.15 (0.89–1.48)	7218	17 (0.24)	1.31 (0.80–2.14)	1.15 (0.66–2.01)
40–44	13,798	95 (0.69)	2.38 (1.85–3.07)	2.39 (1.83–3.12)	2280	7 (0.31)	1.71 (0.81–3.62)	1.04 (0.39–2.80)
45–49	2658	25 (0.94)	3.26 (2.14–4.98)	3.06 (1.96–4.80)	492	4 (0.81)	4.55 (1.69–12.25)	5.05 (1.86–13.66)

^a^ Adjusted for education, occupation, ethnic origin, region, history of using IUD/miscarriage/anemia, menstrual blood loss/BMI/habits on eating meat and eggs.
